# The Characteristic of Transverse Dental Arch Relationship Study in Unilateral Cleft and Palate versus Noncleft in Thai Northeastern Population

**DOI:** 10.1055/s-0044-1795121

**Published:** 2025-04-24

**Authors:** Nattharin Wongsirichat, Araya Pisek, Aggasit Manosudprasit

**Affiliations:** 1 Division of Orthodontics, Department of Preventive Dentistry, Faculty of Dentistry, Khon Kaen University, Khon Kaen, Thailand; 2 Division of Dental Public Health, Department of Preventive Dentistry, Faculty of Dentistry, Khon Kaen University, Khon Kaen, Thailand

**Keywords:** unilateral cleft lip and palate, Angle's classification, overjet, overbite, transverse arch width, modified Huddart/Bodenham index

## Abstract

**Objectives** To evaluate and compare the transverse dental arch relationship of patients with unilateral cleft lip and palate (UCLP) versus the noncleft controls in Northeastern region of Thailand.

**Materials and Methods** A cross-sectional study involving 80 participants comprising 40 nonsyndromic children with UCLP and 40 healthy noncleft children of similar age (mean age: 11 years). The study was conducted at the Faculty of Dentistry, Khon Kaen University, Thailand. Each participant underwent assessments for Angle's classification, overjet, overbite, and transverse dental arch width, along with the evaluation of the modified Huddart/Bodenham (MHB) index using three-dimensional digital dental casts. Comparisons between groups were performed using paired sample *t* -tests and nonparametric Mann–Whitney tests, with a significance level established at *p* < 0.05.

**Results** The sample was categorized into two groups: the early mixed dentition group (ages 7–10 years) and the late mixed dentition group (ages 11–14 years). In the antero-posterior plane, individuals with UCLP and those without cleft presented with class II Angle's classification. In the transverse plane, the maxillary transverse arch width was notably narrower in UCLP cases compared with noncleft cases, with more pronounced differences observed in the late mixed dentition group. However, there was no statistically significant difference in mandibular transverse width between the two groups. The mean MHB index scores were −9.35 in the early mixed dentition group and −12.63 in the late mixed dentition group, indicating a more severe score in the latter. When compared with the noncleft control group, both UCLP groups showed significantly lower MHB index scores.

**Conclusion** In comparison to noncleft individuals, the majority of UCLP cases exhibited class II angles with negative overjet. A significantly smaller transverse arch width was observed in the maxilla of UCLP patients, with no significant variances noted in the mandible. Analysis using the MHB index indicated greater total arch constriction in UCLP cases, particularly in the anterior tooth region. Furthermore, the severity of these findings was observed to escalate with age.

## Introduction

Cleft lip and palate (CLP) is a common craniofacial malformation. 1 The etiology of the defect is multifactorial. Patients with CLP have problems with dental, skeletal, aesthetic, and functional discrepancies. 2 3 Maxillary arch dimension is reduced in these patients due to congenital abnormality or a result of the surgical correction of the primary defects. A reduced transverse arch width influences the occlusion; the most common malocclusion in a patient with CLP is crossbite, especially on the cleft site. 4 5 Unilateral CLP (UCLP) is one of the most prevalent types of orofacial clefts. UCLP results from the failure of fusion between the medial nasal prominence and the maxillary prominence on one side during embryonic development. The malocclusion associated with UCLP is a complex issue from both the primary cleft defect and the secondary effects of surgical interventions. Common dental and skeletal abnormalities in UCLP patients include maxillary arch constriction, posterior crossbite, skeletal class III relationship, rotation and displacement of teeth, arch asymmetry, and alveolar bone deficiency.

To assess the treatment outcome regarding arch dimensions and occlusion after various treatment protocols, different methods have been proposed. CLP indices were developed to evaluate the occlusal relationship in different plane directions. In 1964 the study of Pruzansky and Aduss 6 and in 1970 the study of Matthews et al 7 assessed arch relationship, arch length, and arch width for the examination of crossbite. The other CLP indices are used to determine three dimensions that can be assessed by using the study of GOSLON in1987, 8 the study of GOAL in 1991, 9 and in 1997 of Five-year-old yardstick 10 and bilateral CLP yardstick in 2011 11 was used for bilateral CLP patient. For the index which focuses on assessing transverse dental arch problem, Huddart and Bodenham in 1972 12 developed a numerical scoring system of the crossbite in deciduous dentition and estimated the overall degree of malocclusion. In 2003, Gray and Mossey 13 proposed a modified Huddart/Bodenham (MHB) index to apply to the mixed dentition with an improved scoring system. This index is helpful in locating areas of problematic crossbite at the incisal, canine, premolar, and molar positions. 14 It is the most sensitive and objective index, although this index does not account for anteroposterior skeletal and vertical discrepancies or incisor inclinations, it is an ideal index for assessing transverse discrepancy in CLP recommended by World Health Organization. 15

Proper interpretations of arch form are essential for orthodontic treatment planning like arch expansion, extraction, and aligning. Since the transverse relationship in Thai population with CLP has not yet been assessed, this study aimed to (1) assess the transverse dimension and transverse dental arch relationship using the MHB index in a patient with UCLP, and (2) compare the dimension with those of age-matched and sex-matched noncleft controls.

## Materials and Methods

This study employed a comparative cross-sectional study design conducted from 2021 to 2023. A total of 40 patients diagnosed with UCLP were recruited from the patient registry of the orthodontics clinic at the Faculty of Dentistry, Khon Kaen University, Thailand. The control group consisted of 40 healthy individuals without CLP, who attended routine check-ups at the pedodontic clinic, Faculty of Dentistry, Khon Kaen University. Their ages range between 7 and 14 years with typical growth patterns and maturation without evidence of other craniofacial syndromes or systemic diseases that could have confounded our findings. The inclusion criteria of UCLP were children of both genders with history of cheiloplasty and palatoplasty without any orthodontic intervention. Children with lack of parental consent, noncompliant, and presence of confounding congenital anomalies were excluded from the study.

### Sample Size Calculation

The sample size estimation was done using G power software (Version 2.1.9.2; Faul et al, 2007 33 ) based on the study results of Baraka et al. 16 For a two-tailed dependent sample *t* -test with an effect size *d* = 0.5, α = 0.05, and power (1 − β) = 0.80, the required sample size was 42 (21 per group). This study employed 40 sex- and age-matching between the case and control groups. Specifically, for each participant in the case group, we selected a control participant of the same sex and within ±1 years of age.

### Measurements

The measurements were conducted by a researcher who underwent training for calibration under the guidance of a board-certified orthodontic professional to establish satisfactory inter- and intra-examiner consistency in the assessment of dental arch dimension. Intraclass correlation coefficient (ICC) was used both intra- and inter-examiner reliability. For intra-examiner reliability assessment, each examiner conducted duplicate measurements on 16 subjects, with a 2-week interval between measurement sessions. Inter-examiner reliability was assessed by comparing measurements obtained by the orthodontic professional and researcher on the same cohort of 16 subjects. ICC calculations were performed using SPSS version 26.0 (IBM Corp., Armonk, New York, United States). The resultant ICC scores exceeded 0.8, indicating excellent agreement and reliability of the measurement protocol.

**Fig. 1:**
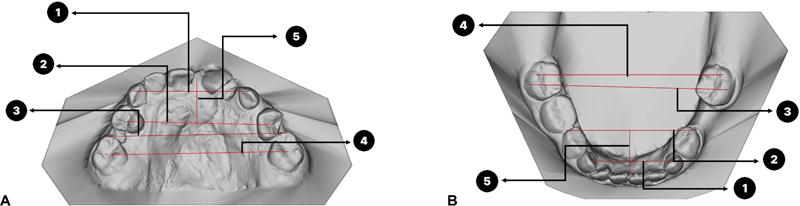
This figure depicts the measurement values for dental arch analysis as follows: (1) inter-canine width (ICW), (2) anterior arch width (AAW), (3) intermolar width (IMW), (4) posterior arch width (PAW), and (5) anterior arch height (AAH) in both the maxilla ( **A** ) and mandible ( **B** ) arches.

The subjects' dental stone models were scanned into three-dimensional (3D) digital cast with 3Shape E2 laboratory scanner (3shape, Copenhagen, Denmark) and were measured using Orthoanalyzer software (3Shape, Copenhagen, Denmark). Dental arch analysis data include the anterior arch width (AAW), posterior arch width (PAW), inter-canine width (ICW), intermolar width (IMW), anterior arch height (AAH), overjet (OJ), and overbite (OB), with detailed definitions for each measurement in the maxilla: (1) ICW of maxilla is the distance from cusp tip of canine to another side; (2) AAW of the maxilla is the distance from the central groove of first premolar to another side in permanent dentition and distal pit of primary first molar to another side in mixed dentition; (3) IMW of maxilla is the distance from the mesiobuccal cusp of the first permanent molar to another side; (4) PAW of maxilla is the distance from the central pit of first permanent molar to another side; (5) AAH of the maxilla is the distance in horizontal plane from the AAW line at mid-palatal suture to the contact point between the maxillary central incisors. Detailed definitions for measurement in the mandible are: (1) ICW of mandible is the distance from cusp tip of canine to another side; (2) AAW of mandible is the distance from buccal contact point of first and second premolar to another side in permanent dentition; (3) IMW of mandible is the distance from the mesiobuccal cusp of the first permanent molar to another side; (4) PAW of mandible is the distance from distobuccal cusp of first permanent molar to another side; (5) AAH of mandible is the distance in horizontal plane from the AAW line to the contact point between the mandibular incisor OJ in class III malocclusion was measured from the most severe in negative OJ (comparing between both incisors) while class II malocclusion was measured from the most severe in positive OJ (comparing between both incisors) 13) OB is measured in overlapping in vertical plane as shown in Fig. 1 .

For assessing sagittal occlusion, the molar relationship was classified into three categories based on Angle's classification: class I, class II, and class III.

### Modified Huddart/Bodenham Index

**Fig. 2:**
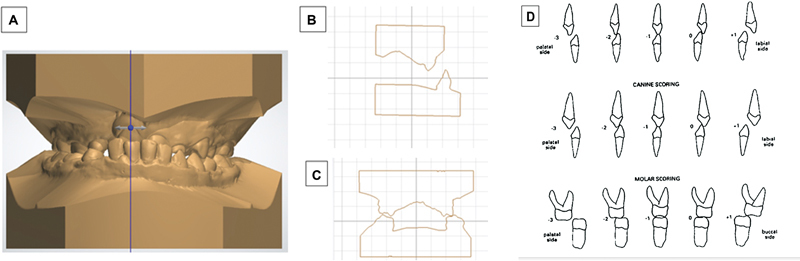
( **A** ) 3D model of patient with unilateral cleft lip and palate. ( **B** ) Assessment of the cross-sectional digital model of the anterior teeth. ( **C** ) Assessment of the cross-sectional digital model of the posterior teeth. ( **D** ) The modified Huddart/Bodenham scoring system. (Adapted from Gray and Mossey 2005 13 .)

In a study aimed at evaluating the prevalence and severity of transverse malocclusions in various CLP conditions, Gray and Mossey 13 introduced the MHB index. This index assesses the presence and extent of crossbites by examining three specific segments: the labial segment, greater (noncleft), and lesser (cleft) buccal segments. Utilizing a five-point continuous scale per tooth with values ranging from −3 to +1, the MHB index yields a total score that can vary between −30 and +10 when summing individual tooth scores. Consequently, the comprehensive grading system involves a detailed 40-point assessment using cross-sectional digital model as shown in Fig. 2 .

### Statistical Analysis

In this study, patient identifiers served as the basis for acquiring demographic and clinical data. The obtained information was input into an IBM SPSS Statistics (Version 28.0) platform for computational analysis. To characterize the dataset, descriptive statistical methods were employed, encompassing variables such as age, gender, laterality of cleft lip or palate, and molar relationships. For evaluating differences in transverse dental arch width and MHB scores between the two groups, paired sample *t* -tests and nonparametric Mann–Whitney tests were applied.

## Results

The research involved 80 participants, with 40 presenting UCLP and 40 serving as noncleft matched controls. The age range was between 7 and 14 years, and the mean age (± SD [standard deviation]) was 10.7 (± 1.73) years in children with UCLP and 11.23 (± 1.82) years in noncleft children. Both groups had 20 males and 20 females per group. In the UCLP group, there were 19 cases with right UCLP and 21 cases with left UCLP. The right side showed an Angle's classification of 27.5% class I, 42.5% class II, and 30% class III, while the left side showed 42.5% class I, 37.5% class II, and 20% class III. In the noncleft group, 37.5% were classified as class I, 47.5% as class II, and 15% as class III. The left side showed 55% class I, 27.5% class II, and 17.5% class III, as detailed in Table 1 . Furthermore, participants were categorized into early mixed dentition (ages 7–10 years) and late mixed dentition (ages 11–14 years) for analysis in accordance with dental developmental stages.

**Table 1:** Demographic data of patients with unilateral cleft lip and palate (UCLP) group and noncleft group

Variable(s)			UCLP number (%)	Noncleft number (%)
**Age (years) 7–14 (** ***n*** ** = 80)**				
** Age (years)**		Mean	10.70 (+1.73)	11.23 (+ 1.82)
** Sex**		Male	20 (50%)	20 (50%)
		Female	20 (50%)	20 (50%)
** Type of cleft**		Left side	21 (52%)	–
		Right side	19 (48%)	–
** Angle's classification**	Class I	Right	11 (27.5%)	15 (37.5%)
		Left	17 42.5%	22 (55%)
	Class II	Right	17 (42.5%)	19 (47.5%)
		Left	15 (37.5%)	11 (27.5%)
	Class III	Right	12 (30%)	6 (15%)
		Left	8 (20%)	7 (17.5%)
**Age (years) 7–10 (** ***n*** ** = 38)**				
** Age (years)**		Mean	9.21 (± 0.89)	9.68 (± 0.85)
** Sex**		Male	10 (52.63%)	10 (52.63%)
		Female	9 (47.36%)	9 (47.36%)
** Type of cleft**		Left side	9 (47.36%)	–
		Right side	10 (52.63%)	–
** Angle's classification**	Class I	Right	8 (42.10%)	4 (21%)
		Left	11 (57.89%)	6 (31.57%)
	Class II	Right	9 (47.36%)	8 (42.10%)
		Left	5 (26.31%)	8 (42.10%)
	Class III	Right	2 (10.52%)	7 (36.84%)
		Left	3 15.78%	5 (26.31%)
**Age (years) 11–14 (** ***n*** ** = 42)**				
** Age (years)**		Mean	12.05 (± 1.07)	12.62 (± 1.20)
** Sex**		Male	10 (47.61%)	10 (47.61%)
		Female	11 (52.38%)	11 (52.38%)
** Type of cleft**		Left side	12 (57.14%)	–
		Right side	9 (42.85%)	–
** Angle's classification**	Class I	Right	7 (33.33%)	7 (33.33%)
		Left	11 (52.38%)	11 (52.38%)
	Class II	Right	10 (47.61%)	9 (42.85%)
		Left	6 (28.57%)	8 (38.09%)
	Class III	Right	4 (19.04%)	5 (23.80%)
		Left	4 19.04%	2 (9.52%)

### Early Mixed Dentition Group (7–10 Years)

The group included a total of 38 children aged 7 to 10 years, with 19 children having UCLP matched with 19 noncleft children. The mean age (±SD) was 9.21 (± 0.89) years for children with UCLP and 9.68 (± 0.85) years for noncleft children, with almost an equal distribution of male ( *n* = 10) and female ( *n* = 9) in both groups. In the UCLP group, there were 10 cases of right UCLP and 9 cases of left UCLP. The molar classification in cleft patients predominantly showed class I and class II malocclusions, with the right side having 42.10% class I, 47.36% class II, and 10.52% class III, while the left side had 57.89% class I, 26.31% class II, and 15.78% class III malocclusions. Conversely, noncleft patients mostly exhibited class II malocclusions at this age, with the right side showing 21% class I, 42.10% class II, and 36.84% class III, and the left side showing 31.57% class I, 42.10% class II, and 26.31% class III.

#### Transverse Dental Arch Width

In the 7- to 10-year-old age group, individuals with UCLP had mean (± SD) measurements for maxillary ICW, AAW, IMW, PAW, and AAH of 30.42 (± 4.24) mm, 34.33 (± 5.73) mm, 49.72 (± 4.38) mm, 42.61 (± 10.07) mm, and 9.75 (± 2.17) mm, respectively. Conversely, noncleft individuals in the same age group had different average measurements for these dental parameters. The mean values for maxillary ICW, AAW, IMW, PAW, and AAH in noncleft children were 34.20 (± 1.73) mm, 38.76 (± 5.64) mm, 52.03 (± 4.15) mm, 45.54 (± 6.47) mm, and 15.90 (± 8.81) mm, respectively. Significant statistical variances were observed between the two groups regarding maxillary ICW ( *p* -value of 0.016), AAH ( *p* -value of 0.002), and OJ ( *p* -value of <0.001) measurements. However, no statistical differences were found in any of the mandibular dental parameters measured as shown in Table 2 . This indicates that the mandibular growth in individuals with UCLP compared with noncleft individuals was quite similar.

**Table 2:** Comparison of dental arch analysis between unilateral cleft lip and palate (UCLP) and noncleft individuals aged 7–10 and 11–14 years

	UCLP		Noncleft		*p* -Value
	Mean	SD	Mean	SD	
**Age between 7 and 10 years**					
**Maxilla (mm)**					
** Inter-canine width (ICW)**	30.42	4.24	34.20	1.73	0.016*
** Anterior arch width (AAW)**	34.33	5.73	38.76	5.64	0.53
** Intermolar width (IMW)**	49.72	4.38	52.03	4.15	0.20
** Posterior arch width (PAW)**	42.61	10.07	45.54	6.47	0.30
** Anterior arch height (AAH)**	9.75	2.17	15.90	8.81	0.002*
**Mandible (mm)**					
** Inter-canine width**	27.02	10.46	26.78	2.60	0.93
** Anterior arch width (AAW)**	34.81	2.13	34.95	2.98	0.87
** Intermolar width (IMW)**	52.84	2.87	52.18	2.80	0.41
** Posterior arch width (PAW)**	49.45	3.03	49.93	3.89	0.67
** Anterior arch height (AAH)**	11.98	1.49	12.61	2.25	0.28
**Vertical and sagittal**					
** Overjet (OJ)**	−4.86	6.15	3.17	0.89	<0.001*
** Overbite (OB)**	3.73	3.33	3.31	2.00	0.69
**Age between 11 and 14 years**					
**Maxilla (mm)**					
** Inter-canine width (ICW)**	30.58	4.11	35.27	2.74	<0.001*
** Anterior arch width (AAW)**	32.22	3.42	37.44	3.23	<0.001*
** Intermolar width (IMW)**	50.68	3.28	53.29	4.27	0.02*
** Posterior arch width (PAW)**	44.89	7.63	48.53	3.74	0.06
** Anterior arch height (AAH)**	10.51	2.74	14.66	2.62	<0.001*
**Mandible (mm)**					
** Inter-canine width**	28.00	2.38	25.44	8.43	0.35
** Anterior arch width (AAW)**	35.77	3.11	35.87	3.06	0.90
** Intermolar width (IMW)**	52.70	3.20	50.12	11.87	0.33
** Posterior arch width (PAW)**	49.64	3.54	49.35	3.13	0.80
** Anterior arch height (AAH)**	13.66	1.66	13.29	1.90	0.43
**Vertical and sagittal**					
** Overjet (OJ)**	−2.88	5.17	3.89	3.14	<0.001*
** Overbite (OB)**	3.96	3.27	3.90	2.19	0.95

Note: Paired sample *t* -test at * *p* < 0.05 (statistical significance).

#### Modified Huddart/Bodenham Index

In the evaluation of the MHB scores in the 7- to 10-year-old age group, it was observed that all variables, except for the molar region in the UCLP group, exhibited a statistically significant narrowing on both the left and right sides of the arch, with a tendency toward greater severity in the anterior region of the arch. A comparison with the noncleft group revealed that the total anterior and premolar regions of the UCLP group displayed a statistically significant constriction, with *p* -values of <0.001 and 0.003, respectively. Additionally, the total arch constriction score in the UCLP group indicated an arch transverse deformity in these patients, with a mean (± SD) score of 12.63 (± 7.04). Furthermore, when compared with the noncleft control group, a statistically significant lower score was evident in the UCLP group, with a *p* -value of <0.001 as shown in Table 3 .

**Table 3:** Comparison of the modified Huddart/Bodenham index between subjects aged 7–10 years and 11–14 years with and without cleft lip and palate

	UCLP			Noncleft		*p* -Value
	Mean	SD		Mean	SD	
**Age between 7 and 10 years**					
**Anterior region**					
** Right central incisor**	−2.47	0.84	0.05	0.91	<0.001*
** Left central incisor**	−2.16	1.25	0.00	0.88	<0.001*
** Right canine**	−1.74	1.24	−0.21	1.08	0.002*
** Left canine**	−1.53	1.43	−0.05	0.78	0.002*
** Total anterior region**	−7.89	3.71	−0.21	3.25	<0.001*
**Premolar region**					
** Right first premolar**	−1.21	1.03	−0.05	0.62	0.002*
** Left first premolar**	−1.00	1.20	−0.16	0.76	0.046*
** Right second premolar**	−0.95	1.08	0.05	0.62	0.003*
** Left second premolar**	−0.79	0.918	0.00	0.75	0.021*
** Total premolar region**	−3.95	3.12	−0.16	2.48	0.003*
**Molar region**					
** Right first molar**	−0.63	0.90	0.05	0.62	0.08
** Left first molar**	−0.16	0.69	−0.26	0.87	0.70
** Total molar region**	−0.79	1.23	−0.21	0.98	0.094
** Total arch constriction**	−12.63	7.04	−0.58	1.38	<0.001*
**Age between 11 and 14 years**					
**Anterior region**					
** Right central incisor**	−1.71	1.30	0.00	1.05	<0.001*
** Left central incisor**	−1.95	1.36	−0.10	1.09	<0.001*
** Right canine**	−0.62	1.24	−0.14	0.91	0.15
** Left canine**	−1.76	1.26	0.00	0.84	<0.001*
** Total anterior region**	−6.05	4.02	−0.23	3.06	<0.001*
**Premolar region**					
** Right first premolar**	−0.71	0.85	0.00	0.84	0.01*
** Left first premolar**	−0.86	1.06	−0.05	−0.69	0.01*
** Right second premolar**	−0.76	0.89	−0.19	0.60	0.02*
** Left second premolar**	−0.81	1.03	−0.14	0.57	0.01*
** Total premolar region**	−3.14	2.69	−0.38	2.52	0.002*
**Molar region**					
** Right first molar**	−0.19	0.60	0.00	0.31	0.16
** Left first molar**	−0.33	0.66	0.00	0.31	0.49
** Total molar region**	−0.52	0.87	0.00	0.45	0.02*
** Total arch constriction**	−9.35	6.12	−0.62	1.17	<0.001*

Note: Paired sample *t* -test at * *p* < 0.05 (statistical significance).

### Late Mixed Dentition Group (11–14 Years)

A total of 42 children aged between 10 to 14 years, comprising equal numbers of UCLP cases and matched controls, the mean ages (± SD) were 12.05 (± 1.07) years for UCLP children and 12.62 (± 1.20) years for noncleft counterparts. Gender distribution included 10 males and 11 females in each group. Notably, there were 9 cases of right UCLP and 12 cases of left UCLP observed. The molar classification in cleft patients predominantly showed class II malocclusion, with the right side having 33.33% class I, 47.61% class II, and 19.04% class III, while the left side had 52.38% class I, 28.57% class II, and 19.04% class III. Similarly, noncleft patients exhibited similar molar classifications on both sides with the right side showing 33.33% class I, (42.85%) class II, and 23.80% class III and the left side showing 52.38% class I, 38.09% class II, and 9.52% class III, respectively.

#### Transverse Dental Arch Width

In the second age group (10–14 years), the average (± SD) measurements in individuals with UCLP were as follows: maxillary ICW 30.58 (± 4.11) mm, AAW 32.22 (± 3.42) mm, IMW 50.68 (± 3.28) mm, PAW 44.89 (± 7.63) mm, and AAH 10.51 (± 2.74) mm. In comparison, for noncleft individuals, the mean (± SD) measurements were: maxillary ICW 35.27 (± 2.74) mm, AAW 37.44 (± 3.23) mm, IMW 53.29 (± 4.27) mm, PAW 48.53 (± 3.64) mm, and AAH 14.6 6(± 2.62) mm. Statistically significant differences were observed between the UCLP and noncleft groups in terms of maxillary ICW, AAW, IMW, and AAH, with most variables showing a *p* -value of <0.001, indicating lower means in the UCLP group compared with the noncleft group, as shown in Table 2 . However, there was no statistical distinction in dental parameters in the mandible when comparing the UCLP and noncleft groups.

#### Modified Huddart/Bodenham Index

The study found that the majority of MHB index variables in the 11- to 14-year group, except for the right canine and left and right molar regions, showed a statistically significant constriction of the arch. The severity of the constriction leaned toward the anterior region of the arch. When compared with the noncleft group, the total anterior, premolar, and molar regions of the UCLP group showed a statistically significant constriction with *p* -values of <0.001, 0.002, and 0.02, respectively. The total arch constriction score of the UCLP group also showed a transverse arch constriction with a mean (± SD) of 9.35 (± 6.12), which was less severe than the younger age group. When compared with the noncleft control, a statistically significant lower score of UCLP was shown with a *p* -value of <0.001 as shown in Table 3 .

#### Superimposition

**Fig. 3:**
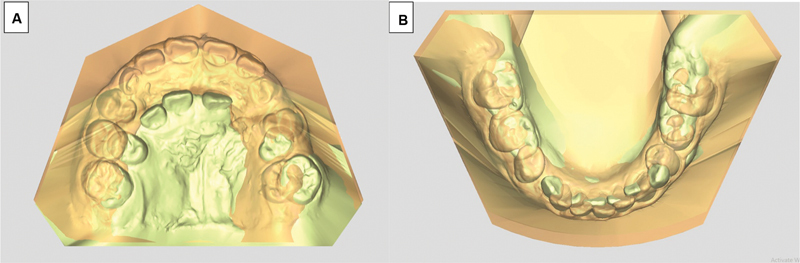
This figure illustrates the comparison of dental arch superimposition between subjects with unilateral complete cleft lip and palate and those with a normal condition, showing the upper arch ( **A** ) and lower arch ( **B** ).

The representative 3D dental casts of UCLP and noncleft patients in the 7- to 10-year group were superimposed using an Orthoanalyzer (3Shape, Copenhagen, Denmark) program, an occlusal reference plane passing through the following point with the mesiobuccal cusp of left and right permanent molar and mid-anterior teeth as shown in Fig. 3 .

## Discussion

Patient with CLP manifest anomalies within all three craniofacial structural dimensions, clearly visible in the transverse and anteroposterior directions. These anatomical abnormalities exert a direct influence on occlusion, marked by apparent maxillary constriction. Additionally, these patients frequently present with dentoskeletal discrepancies, including malocclusions characterized by dental misalignment, anterior and posterior crossbite. 4

This study aimed to examine the transverse dental arch relationships in children with UCLP compared with typically developing children aged 7 to 14 years. The evaluation included Angle's classification, maxillary and mandibular transverse dental arch width, and the MBH index to assess transverse dental relationships. The research cohort consisted of 80 participants, split evenly between the UCLP group and the control group, ensuring age range parity. Furthermore, this study divided the age groups into 7–10 and 11–14 years to analyze transverse dental arch relationships during the early and late phases of mixed dentition. The study on Angle's classification of malocclusion among subjects found that both the control group and the UCLP group exhibited dental class II malocclusion, contrary to previous findings by Paradowska-Stolarz and Kawala. 4 This deviation challenges the established belief that individuals with CLP have class III malocclusion. The prevalence of class II malocclusion in the mixed dentition phase is attributed to environmental factors affecting jaw size, tooth size, and quantity, leading to mesial migration of the upper permanent first molar or ectopic eruption of premolars and canines within a constricted maxilla. 17 18 19 20

In the assessment of transverse dental arch width, a notable distinction was observed in the early mixed dentition phase (7–10 years) between individuals with UCLP and the control group. Specifically, there was a significant difference in ICW between these two groups. Conversely, in the late mixed dentition group (11–14 years), all maxillary measurements, with the exception of PAW, exhibited differences between the UCLP and control groups. This disparity highlighted a more pronounced narrowing of the arch width in children aged 11 to 14 compared with those aged 7 to 10, potentially indicating a stunted growth of the maxilla in the UCLP group as opposed to the continuous growth of the upper jaw in unaffected individuals. The growth of the upper jaw typically ceases with the fusion of the mid-palatal suture, a process that typically concludes around 16 years in females and 18 years in males. 19 21 22 In the early mixed dentition group (7–10 years), Numerous studies corroborate our findings. Generali et al's study evaluated maxillary arch dimensions in individuals with UCLP using 3D maxillary digital models. Their findings indicated that children aged 5 to 12 years with UCLP exhibited a decrease in ICW, while IMW was comparable to noncleft individuals. 23 This aligns with the observations of Baraka et al, who studied children with UCLP aged 4 to 9 years, and Gopinath et al, who examined children with UCLP aged 7 to 13 years, both noting a significant reduction in ICW and a nonsignificant change in IMW compared with controls. 24 25 Conversely, Athanasiou et al's study on children with UCLP aged 3 to 9 years reported reductions in both ICW and IMW, conflicting with this study's findings in the early mixed dentition group regarding PAW measurements. 26 27 In the late mixed dentition phase, research results of Wahaj and Ahmed in adolescents aged 14 to 16 with UCLP aligned with our results, demonstrating significantly reduced ICW and IMW compared with normal control. 27 28 Conversely, Athenasiou et al's 26 study on adolescents aged 12 with UCLP showed reduced ICW while IMW remained similar to noncleft individuals. 27

In the context of the MHB index, individuals diagnosed with UCLP within the early mixed dentition group demonstrated a mean value of −9.35, whereas the control group exhibited a mean value of −0.62. Within the late mixed dentition group, individuals with UCLP displayed a mean total score of −12.63, while the noncleft group showed a mean score of −0.58. These findings align with prior research, 16 29 30 31 32 which reported MHB scores ranging from −7 to −14 in UCLP patients aged 5 to 12 years. Analysis at the individual tooth level revealed a higher prevalence of crossbite deformities at the cleft-sided central incisor, noncleft-sided central incisor, and canines, with fewer severe crossbites observed in the permanent molar region. This pattern is consistent with previous studies. 16 31 Notably, in participants aged 11 to 14, no significant differences were observed in the positioning of right canines compared with the control group, likely influenced by the predominance of left-sided clefts in this study group. Canines adjacent to the cleft side commonly exhibited malpositioning or ectopic eruption, contrasting with normally erupted canines on the noncleft side.

In summary, this study underscores the progressive reduction in transverse maxillary width attributed to scar tissue contraction as individuals with cleft palate age. These findings contribute valuable insights into the dental characteristics and developmental patterns associated with UCLP, highlighting the importance of early intervention and ongoing monitoring in this patient population.

While the MHB index demonstrated effectiveness in evaluating transverse malocclusion problems, its application in patients with UCLP revealed certain limitations, particularly when assessing the anterior tooth region. The index, initially developed for the assessment of the transverse plane, encounters challenges when extended to encompass the anteroposterior direction. Because of this restriction, the authors suggest segmenting this analysis into a section that concentrates solely on the transverse plane. Moreover, the authors of the study acknowledge a specific limitation in the MHB index with regard to its scoring of posterior crossbite. Specifically, this limitation is denoted by a score of −3, which is assigned when there is a lack of occlusion between teeth in the posterior region. The authors caution that the −3 score may not precisely capture the full extent of the posterior crossbite issue. This suggests that relying solely on this score may underestimate the severity and complexity of the malocclusion problem in these patients. To address this potential limitation, the authors recommend the inclusion of additional assessments or supplementary analyses to obtain a more comprehensive understanding of the complexity and extent of malocclusion problems in patient with CLP. By incorporating these supplementary measures, such as radiographic evaluation or 3D imaging techniques, a more accurate and detailed evaluation of the malocclusion can be achieved.

By acknowledging this limitation and suggesting further assessments, the authors aim to encourage a more precise tool for evaluation and management of transverse dental arch discrepancy in those patients.

## Conclusion

In the antero-posterior plane, patients with UCLP showed a tendency toward class II Angle's classification attributed to the mesialization of upper molars. Additionally, a reduction in transverse width was observed in the maxillary arch of these patients with UCLP compared with normal controls, as indicated by transverse arch analysis and the MHB score with no noticeable differences in the mandible. Furthermore, the severity of these findings was noted to increase with age.
